# Short time effects of a low-frequency, high intensity magnetic field in the treatment of chronic neck and low back pain

**DOI:** 10.3934/publichealth.2022021

**Published:** 2022-02-10

**Authors:** Mattia Fortina, Aurelio Vittoria, Stefano Giannotti, Pasquale Biandolino, Gabriele Cevenini, Serafino Carta

**Affiliations:** 1 Orthopedics Department, University Hospital of Siena, Siena, Italy; 2 University of Siena, Siena, Italy; 3 Department of Anesthesia and Cardiothoracic Vascular Intensive Therapy, University Hospital of Siena; Siena, Italy; 4 Medical Biotechnologies Department, University of Siena, Siena, Italy; 5 Orthopedics and Traumatology Department, University Hospital of Siena, Siena, Italy

**Keywords:** PEMF, back pain, neck pain, magnetic-therapy, musculoskeletal disorders, conservative treatment

## Abstract

**Introduction:**

Neck and back pain afflicts millions of people. Magnetotherapy has shown to have anti-inflammatory effects that could act on pain generation, but the literature lacks provide a precise therapeutic protocol.

**Methods:**

A high-intensity electromagnetic field with a dedicated applicator was administered to 38 patients with low-back pain and 30 patients with neck pain. The device provides 60 mT and a frequency of 50 Hz for 30 minutes, the session was repeated 4 times.

**Results:**

The mean VAS of the low-back pain group decreased from 6.56 to 4.54, with a significant reduction of 30.8%. The mean VAS of the neck pain group decreased from 6.51 to 1.96, with a significant reduction of 69.9%.

**Discussion:**

The treatment used showed good results in both groups of the patient, without side effects. The therapeutic protocol adopted is safe, provide rapid relief from the pain and is not time demanding. This treatment could represent an effective non-pharmacologic physical therapy option in the treatment of low-back pain and cervical pain.

## 1. Introduction

Working-age adults often experience musculoskeletal disorders. Neck pain and low back pain are the most common symptoms [1],[2].

These conditions are a significant medical problem and are the principal cause of work absences in industrialized societies [3],[4]. Both result in high costs for national economics and health care systems because of long-lasting and cost-intensive treatments, especially in chronic cases. The therapies proposed include non-invasive and minimally invasive or invasive modalities, which could be associated with high risks of adverse effects and increased morbidity [5]–[7].

A widely used technology for the non-invasive treatment of musculoskeletal disorders is pulsed electromagnetic fields (PEMF). PEMFs are selected low-frequency electromagnetic fields without ionizing or thermal effect [8]. The growing interest in their mechanisms of action has led to numerous in vitro trials confirming their effectiveness in up-regulating anti-inflammatory adenosine receptor A2A, A3, reducing PGE2, and pro-inflammatory cytokine IL-6, IL-8, and in the inhibition of the factor NF-kB transcription [9],[10]. Some authors have documented the biological efficacy and tolerability of PEMF for osteoarthritis [11], shoulder periarthritis [12], neck pain [13] and back pain [14]. However, other researchers failed to found positive results after PEMF application in painful conditions [15]. The wide variability in the results may be due to the differences in the many protocols adopted that used various lengths and sessions. Some devices require daily treatments lasting many hours that in many studies have been disregarded. Also, fundamental parameters like frequency, field strength, pulse rate and width differ significantly between the studies [16]. To improve patient compliance, we adopted a high-intensity low-frequency electromagnetic field transmitted by an applicator with a dedicated shape to cover a large area of the body. This research aims to evaluate the efficacy and the safety of the treatment in an observational study on patients with low back pain and cervical pain.

## 2. Materials and methods

### 2.1. Patients

Sixty-eight subjects (42 females and 26 males), out of 120 patients screened, between 27 and 89 years old were enrolled in this open study: 38 patients (23 females and 15 males) with low back pain, and 30 patients (19 females and 11 males) affected by cervical pain. Inclusion criteria were: neck or back pain since more than three months; degenerative changes of one or more intervertebral disc documented by magnetic resonance imaging in the last year, according to the CTF (Combined Task Force) classification [17] and graded as desiccation, fibrosis, narrowing of the disc space, diffuse bulging of the annulus beyond the disc space, fissuring, mucinous degeneration of the annulus, intra-discal gas, osteophytes of the vertebral apophyses, defects, inflammatory changes, and sclerosis of the endplates; patients without nerve root compression according to the van Rijin classification [18]; previous treatment with analgesic or non-steroidal anti-inflammatory drugs. Exclusion criteria were: disc degeneration classified as “extrusion” by the CTF classification, patients with nerve root compression, patients with prior vertebral surgery, tumors, spinal infections, spinal stenosis or spondylolisthesis, pacemaker, current or previous treatment with analgesic or non-steroidal anti-inflammatory drugs and other physical therapy, assumed within the last six months. Approval for the study was sought from and provided by the local Ethical Committee and the Institutional Review Board. No trial registry number was requested for this kind of study.

**Figure 1.**
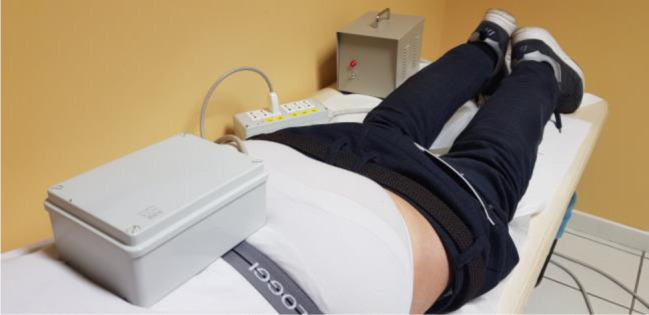
The device applicator positioned on the low-back of a patient.

The treatment with the high-intensity low-frequency electromagnetic field lasted for 30 minutes, was repeated four times with a mean interval of 26 hours (+/−2). The applicator of the device was positioned directly on the painful area (Figure 1).

Each patient quantified the painful symptoms on a Visual Analogue Scale (VAS); the scale was graded from 1 (minimum pain) to 10 (maximum pain intensity). Measurements were taken 30 minutes before the first treatment and 24 hours after the last treatment. No other therapy was performed during the study period. Patients are encouraged to conduct the normal activity of daily living. The only rescue medication approved was Paracetamol 500 mg, and its intake was recorded.

### 2.2. The device

The station consists of two parts. One is connected to the electricity grid (220 V at 50 Hz) and reduces the electric current from 220 V to 60 V. The second part is the electromagnetic fields generator that consists of a coil formed by a winding of enamelled copper wire, with dedicated number of turns N, arranged around the central leg of an E-shaped beam of iron-silicon laminas of the type usually used in transformer consoles. The magnetic circuit is not closed as the complementary side of the transformer joins the three rods of the E only on one side while the other side remains open.

**Figure 2.**
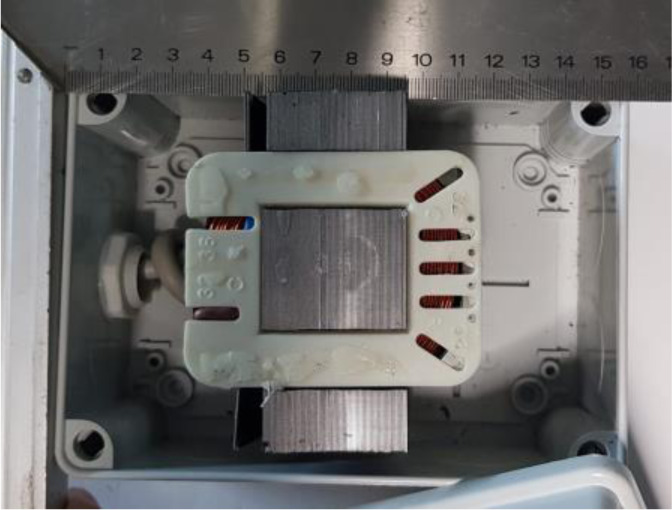
The internal structure of the device.

The polar expansion in the middle measures 33 mm × 38 mm while the side ones, 16 mm distant from it, measure 17 × 38 mm (Figure 2).

The rods of the E act as polar expansions that operate as a triad of electromagnets in which the control unit has always an opposite polarity than the lateral rods. The device label is CE1370.

The winding is powered at 60 V with alternating current at a frequency of 50 Hz, with an intrinsic resistance calculated so that Ohm's law is always satisfied. The intensity of the relative current is 0.8 A. The magnetic flux density or the magnetic induction, measured with a Hall probe (MG-4D Gaussmeter, Walker Scientific inc. Rockdale Street, Worcester, MA, 01606 USA) in contact with the expansion in the middle, is 60 mT. Considering the distinctive geometrical conformation of the rods that generate the field and the consequent spatial distribution of the relative flow lines, the electromagnetic forces induced in the tissues produce activity in a large area of the body.

### 2.3 Statistical analysis

We calculated the VAS score means (±standard deviation) for both groups before and after the treatment. The mean reduction in pre- and post-treatment VAS pain intensity was assessed with the one-tailed Student's t-test for paired data, as the Kolmogorov-Smirnov test excluded a significant deviation from normality of VAS changes. The minimum sample size was determined to have an intermediate Cohen's effect size of 50%, a power of not less than 80% and a significance of 95%. It was 27, properly lower than the smallest group of 30 patients with neck pain.

G*Power software, version 3.1.9.4, and SPSS software, version 10, (SPSS Inc., Chicago, IL, USA) were used to perform sample size analysis, and descriptive and inferential statistical analyses, respectively. A p-value <0.05 was assumed to assess the significance of all statistical testing.

## 3. Results

The affected intervertebral disc and the recorded pain perceived before and after the treatment according to a visual analogue scale are summarized in Tables 1 and 2, for each subject who participated in the study. No patient was found as a non-responder presenting no change of VAS or worsening of the VAS score. Appendix 1 reported the consort statement flow chart.

In Table 1 we have summarized the thirty-eight patients suffering from low back pain. They had a basal VAS higher than the cervical group. The pre-treatment VAS (mean 6.56) decreases to a mean value of 4.54 after four applications; this pairwise VAS reduction was statistically significant (p < 0.001), equal to 30.8%.

**Table 1.** Personal data, pathology level and VAS assessment of pain intensity before and after the four treatments with Magnetic Fields in the group of 38 patients (15 males and 23 females) with low back pain.

Anagraphic Data	Age (Sex)	Pathology Level	Basal Pain Intensity according to VAS score	Post Pain Intensity according to VAS score	Difference in Pain Intensity according to VAS score
P.E.	29 (M)	L5–S1	6	1	5
M.D.	60 (F)	L4–S1	4	2	2
F.A.	32 (M)	L4–S1	7	1	6
V.S.	57 (F)	L3–L4	6	2	4
T.U.	79 (M)	L5–S1	5	3	2
S.F.	83 (F)	L3–L4–L5–S1	6	4	2
L.S	89 (F)	L4–L5–S1	5	2	3
B.B	54 (F)	L3–L4–L5	7	2	5
B.A.	67 (F)	L5–S1	6	3	3
S.S.	68 (F)	L5–S1	4	1	3
B.S.	86 (F)	L4–L5S1	5	3	2
J.A.V.	27 (M)	L4–L5	7	2	5
S.S.	87 (F)	L5–S1	6	1	5
R.G.	74 (F)	L5–S1	5	1	4
L.C.	51 (F)	L4–L5–S1	6	1	4
M.R.	62 (M)	L4–S1	7	1	5
G.F.	51 (F)	L5–S1	6	3	3
T.U.	79 (M)	L1–S1	7	4	3
D.J.P.	67 (F)	L5–S1	4	2	2
R.G.	77 (F)	L1–S1	5	1	4
F.M.	71 (M)	L2–S1	6	2	4
F.R.	76 (F)	L3–L4	5	2	3
R.S.	45 (F)	L4–L5	6	1	5
F.V.	67 (F)	L4–L5	6	4	2
I.A.	69 (M)	L3–L4	5	1	4
B.A.	69 (M)	L5–S1	6	2	3
M.V.	71 (F)	L5–S1	6	1	5
B.L.	52 (F)	L5–S1	7	2	5
D.S.I.	71 (F)	L5–S1	8	3	5
Y.L.S.	55 (M)	L5–S1	8	2	6
V.S.	57 (F)	L5–S1	5	3	2
C.G.	65 (F)	L4–L5–S1	7	5	2
M. A	69 (M)	L2–L3–L4	8	3	5
G. M.	40 (M)	L5–S1	8	7	1
S. V.	77 (F)	L5–S1	8	7	1
A. E.	87 (M)	L1–S1	8	5	3
B. R.	52 (M)	L2–L5	8	6	2
D. E. A.	89 (M)	L2–S1	8	6	2
Means ± S. D.	64.7 ± 16.3		6.237 ± 1.240	2.684 ± 1.741	3.474 ± 1.409
P value for paired data			**	**	**

In Table 2 we have synthesized the thirty patients with neck pain: their symptomatology responds promptly to the electromagnetic treatment; the mean pre-therapy value of the VAS score decreases significantly from 6.51 to 1.96 after the treatment. The result was statistically significant (p < 0.001). The mean pairwise VAS was reduced by 69.9%.

**Table 2.** Personal data, pathology level and VAS assessment of pain intensity before and after the four treatments with Magnetic Fields in the group of 30 patients (11 males and 19 females) with neck pain.

Anagraphic Data	Age (Sex)	Pathology Level	Basal Pain Intensity, according to VAS score	Post Pain Intensity according to VAS score	Difference in Pain Intensity according to VAS score
B.P.	61 (M)	C5–C6	8	2	5
M.R.	68 (F)	C3–C7	8	2	2
R.G.P.	71 (M)	C3–C7	8	2	6
S.F.	83 (F)	C3–C7	8	2	4
B.A.	67 (F)	C6–C7	6	2	2
S.S.	78 (F)	C3–C7	5	1	2
B.S.	87 (F)	C3–C4	6	2	3
G.F.	51 (F)	C4–C5	7	3	5
R.F.	76 (F)	C3–C4–C6–C7	8	2	3
S.R.	82 (F)	C6–C7	6	2	3
B.A.	69 (M)	C3–C6	7	1	2
F.F.	43 (M)	C4–C5	6	2	5
L.M.	39 (M)	C3–C4–C5–C6	7	1	5
A.V.	85 (F)	C4–C7	8	4	4
P.G.	72 (M)	C5–C7	6	2	4
P.F.	66 (F)	C4–C7	8	2	5
M.J.E.	50 (F)	C5–C7	6	1	3
R.D.	65 (M)	C4–C7	6	1	3
G.R.	60 (F)	C5–C7	6	1	2
M.L.	81 (M)	C4–C7	5	2	4
A.B.	69 (M)	C4–C7	6	1	4
L.P.L.	52 (M)	C5–C7	6	2	3
G.S.	73 (M)	C6–C7	5	3	5
P.E.	54 (F)	C6–C7	6	1	2
B.M.	68 (F)	C4–C7	5	1	4
P.S.	47 (F)	C1–C5	6	2	3
B.R.	61 (F)	C3–C4	6	1	5
B.I.	82 (F)	C5–C7	8	1	5
V.S.	57 (F)	C5–C7	6	2	5
M.A	69 (F)	C5–C6–C7	6	2	4
Means ± S. D.	66.2 ± 12.8		6.51 ± 0.99	1.96 ± 0.91	4.48 ± 1.12
P value for paired data			**	**	**

All patients in the two groups, low back and cervical pain, reported a significant and rapid improvement in subjective pain. Three subjects in the cervical and two in the low back groups took one tab of the rescue medication (paracetamol) after the first session of the electromagnetic fields.

Every subject of both groups completed the short therapeutic cycle without side effects that may have required suspension or cessation of the treatment.

## 4. Discussion

Musculoskeletal system disorders (MSDs) are the most commonly encountered disease in orthopaedics and physiotherapy practice in the world. Low back pain, in particular, is the most prevalent presentation with long abstention from work and high economic losses. Chronic cases with a pain history longer than 12 weeks are the most difficult to treat. Therefore, it is crucial to establish new effective and nonsurgical treatment modalities that guarantee minimal side effects.

Our observational study provides reliable evidence that a high-intensity electromagnetic field with a dedicated probe is very effective for neck and low back pain treatment. Each patient in the present open clinical study reported a prompt, rapid and almost complete regression of local pain within four sessions of 30 minutes each.

The magnetic field that we have applied to our group of patients has been able to interfere with the pro-inflammatory mechanisms in the appearance and persistence over time of the painful symptoms. The effects of the electromagnetic field on the inflammatory cascade were previously studied with the use of fixed frequency magnetic fields (ELF) and with variable over time magnetic fields (TAMMEF) also in other diseases characterized by chronic pain [11]–[14],[19],[20]. Several in vitro trials documented that known biochemical pathways are stimulated by electromagnetic impulses, including stimulating osteoblast growth activity [14], neovasculogenesis, the release of growth factors [21] and improvement of blood supply. In this context, clinical trials showed an increase in tissue blood flow in the lumbo-pelvic region and improved lumbo-pelvic stability after core training among patients with chronic non-specific low back pain [22].

Moreover, the non-invasiveness and well-tolerability of our technique made it applicable in a vast cohort of patients, as demonstrated by the great age range of the people that we had treated, between 27 and 87 years. Even patients with co-morbidities like gastric, hepatic or renal pathologies, have been safely treated.

This study has some limitations. It lacks a control group with a shame therapy, but the positive effects of magnetotherapy with validated parameters are well documented, hence we haven't found it productive to divide patients. Another bias is the absence of a longer follow-up to evaluate the possible relapses. Further studies are needed to assess also the impact of the therapy on physical activity and the activity of daily living.

## 5. Conclusions

In conclusion, these results suggest that the treatment with high-intensity low-frequency magnetic fields reduces pain and disability in patients suffering from non-specific low back pain and cervical pain. This treatment could represent an effective non-pharmacologic physical therapy option for these patients, but prospective controlled blinded trials are needed to confirm and validate our results.
